# A Novel Small Molecule Enhances Stable Dopamine Delivery to the Brain in Models of Parkinson’s Disease

**DOI:** 10.3390/ijms26094251

**Published:** 2025-04-30

**Authors:** Xiaoguang Liu, Michaeline L. Hebron, Max Stevenson, Charbel Moussa

**Affiliations:** Translational Neurotherapeutics Program, Laboratory for Dementia and Parkinsonism, Department of Neurology, Georgetown University Medical Center, Washington, DC 20007, USA; mlh88@georgetown.edu (M.L.H.); mes407@georgetown.edu (M.S.)

**Keywords:** Pegasus, tetracycline, dopamine, levodopa, Parkinson’s disease

## Abstract

Levodopa is the gold standard symptomatic treatment for Parkinson’s disease. Disease progression due to alpha-synuclein accumulation, brain inflammation, and the loss of dopamine neurons, as well as motor fluctuations, due to variations in levodopa plasma levels, remain a significant problem for Parkinson’s patients. Developing a therapeutic option that can simultaneously reduce the neuropathology associated with alpha-synuclein aggregation, attenuate oxidative stress and inflammation, and overcome variations in levodopa plasma levels is an unmet need to treat Parkinson’s disease. We determined the pharmacokinetics and pharmacodynamics of a small molecule, dubbed Pegasus, that conjugates dopamine with a nonantibiotic doxycycline derivative via a molecular linker. Mice harboring the human A53T mutation of alpha-synuclein or treated with MPTP were injected once daily with 50 mg/kg Pegasus for 2 weeks and assessed for motor, behavioral, and cognitive effects, followed by biochemical and histochemical analysis. Pegasus is a poor brain penetrant but it was metabolized to stable dopamine and tetracycline derivatives, and abundant plasma and brain levels of these metabolites were detected. Pegasus reduced soluble and insoluble alpha-synuclein levels, protected dopamine-producing neurons, and reduced astrocytic activation in A53T mice. Mice treated with Pegasus exhibited motor improvement (6.5 h) and reduction in anxiety-like behavior. Rotarod and grip strength improved in MPTP-treated mice when mice were treated with Pegasus or levodopa. Pegasus may be a multi-modal therapeutic option that can deliver stable dopamine into the CNS and reduce misfolded alpha-synuclein, activate dopamine receptors, and attenuate variations in dopamine levels.

## 1. Introduction

Parkinson’s disease (PD) is a neurodegenerative disorder causing motor and non-motor symptoms, the loss of dopamine (DA) in neurons of the nigro-striatal pathway, and the accumulation of misfolded alpha-synuclein [1,2,3]. Levodopa is the gold standard treatment for PD symptoms, but progression due to alpha-synuclein accumulation, pathological oxidative stress, brain inflammation, and the loss of DA neurons are not abated by levodopa. Motor fluctuations, due to variations in levodopa absorption, levodopa plasma levels, and brain penetration remain significant problems for patients. Optimizing a therapeutic option that can simultaneously halt alpha-synuclein aggregation and overcome variations in levodopa plasma levels is an medical need to treat PD [4,5,6].

A novel compound dubbed Pegasus was synthesized [7] and shown to address the multifactorial cause of PD, including the aggregation of alpha-synuclein, oxidative stress, and inflammation in vitro [7]. Pegasus is a small molecule (with a molecular weight of 652 Da) that links DA with a nonantibiotic doxycycline derivative [7], resulting in a molecule with calculated physicochemical properties not predicted to cross the blood–brain barrier (BBB) via passive permeability [8]. Here, we report novel mechanisms of DA delivery into the brain via the in vivo metabolism of Pegasus into DA and tetracycline/doxycycline derivatives to provide significantly enhanced DA delivery upon peripheral dosing. Treatment with 1-methyl-4-phenyl-1,2,3,6-tetrahydropyridine (MPTP) causes motor symptoms reminiscent of PD, while Pegasus can restore behavior like levodopa in vivo.

## 2. Results

### 2.1. Tolerated Dose and Tissue Toxicity

**Figure 1:**
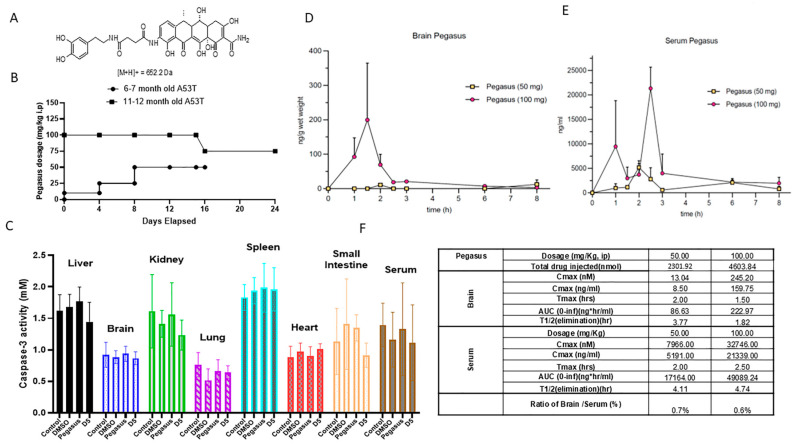
Toxicity and pharmacokinetic study of Pegasus. (**A**) Chemical structure of Pegasus. (**B**) Administration of Pegasus revealed maximum tolerable dose and in vivo toxicity verified by (**C**) caspase-3 activity. Pharmacokinetic analysis of Pegasus in (**D**) brain and (**E**) serum and (**F**) calculated pharmacokinetic parameters. One-way ANOVA with Dunnett’s multiple comparisons test. N = 3–10. All values presented as mean ± SD.

Pegasus synthesis and the D5 control derivative of tetracycline were previously described [7]. Pegasus is a 652.2 Da small molecule that consists of one DA conjugated to tetracycline via a molecular linker (Figure 1A). Extensive in vitro studies were previously performed and showed Pegasus’s protective effects via the prevention of alpha-synuclein misfolding [7]. We determined a tolerated dose of Pegasus in young (6–7 months old) and old (11–12 months old) TgA53T mice (IP, once daily) treated with an ascending dose of Pegasus of 10 mg/kg, 50 mg/kg, and 75 mg/kg for 4 consecutive days (Figure 1B). Pegasus was tolerated up to 75 mg/kg without visible adverse effects. However, at 100 mg/kg, male and female (12–13 months old) mice showed some scruffiness and weakness at Day16, and one mouse showed gut toxicity at autopsy. We then reduced the dose to 75 mg/kg for another week. Autopsy was performed and no adverse effects were detected.

We further verified cell death via caspase-3 activity assays of the liver, brain, kidney, lung, spleen, heart, serum, and small intestine in young TgA53T mice (6–7 months old). There were no significant changes in caspase-3 activity in all the tested tissues in Pegasus-treated mice compared to naïve control (without any treatment), DMSO-, and D5-treated mice (Figure 1C).

### 2.2. Pharmacokinetics

PK analysis showed that Pegasus could be detected in both the serum and the brain after a single intraperitoneal (i.p.) injection (Figure 1D,E) in wild-type mice. Pegasus reached peak concentration at 2 h after an injection of 50 mg/kg in both the serum and the brain, and reached peak concentration at 1.5 h in the brain and 2.5 h along with an additional smaller peak at 1 h in the serum after an injection of 100 mg/kg i.p. AUC_0-inf_ was 86.63 to 222.97 ng × h/mL in the brain and 17,164 to 49,089.24 ng × h/mL in the serum. The elimination half time T_1/2_ of Pegasus was 4.11 to 4.74 h in the serum and 1.82 to 3.77 h in the brain. The percentage ratio of brain/serum was 0.6–0.7% (Figure 1F).

**Figure 2:**
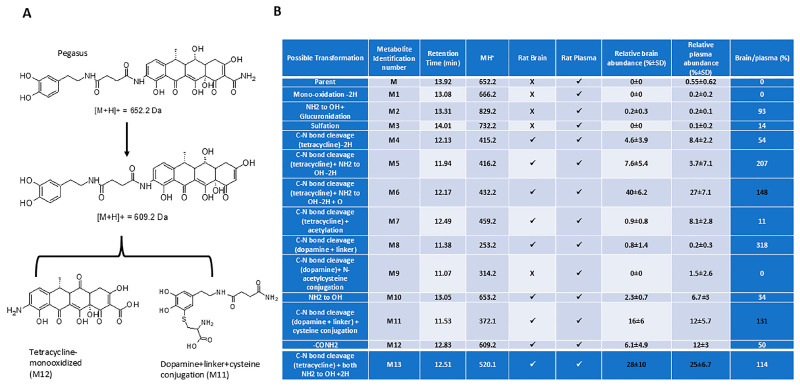
In vivo metabolism of Pegasus. (**A**) Possible metabolic pathways for Pegasus and matrix differences in metabolite formation and (**B**) relative abundance of metabolites of Pegasus in rat brain and plasma samples. All values presented as mean ± SD.

In vivo metabolism: We performed a metabolism study (Figure 2) in male Sprague Dawley rats (6–8 weeks old) after a single i.p. injection of 50 mg/kg Pegasus. Plasma and brain samples were serially collected and assessed for the presence of possible phase-I and phase-II metabolites. A total of thirteen metabolites were detected in the rat brain and plasma samples. They were identified as formed by the mono-oxidation -2H (M1), NH2 to OH + glucuronidation (M2), sulfation (M3), C-N bond cleavage (tetracycline) -2H (M4), C-N bond cleavage (tetracycline) + NH_2_ to OH -2H (M5), C-N bond cleavage (tetracycline) + NH_2_ to OH -2H + O (M6), C-N bond cleavage (tetracycline) + acetylation (M7), C-N bond cleavage (dopamine + linker) (M8), C-N bond cleavage (dopamine) + N-acetylcysteine conjugation (M9), NH_2_ to OH (M10), C-N bond cleavage (dopamine + linker) + cysteine conjugation (M11), -CONH_2_ (M12), and C-N bond cleavage (tetracycline) + both NH2 to OH + 2H (M13) of parent (M). The parent peak (Figure 2B) was very small in the rat plasma samples and was not detected in the brain samples. The possible metabolic pathways for Pegasus are presented in Figure 2A and Supplemental Figure S1. The [M + H] + (Q1) with retention time and product ions (MS/MS, fragmentation pattern) for the parent Pegasus and its putative metabolites, as well as the differences in the observation of the metabolites in the rat brain or plasma are shown in Figure 2B. The relative abundance of Pegasus and its metabolites in the brain and plasma was determined at the ionization parameters of the parent. The major metabolite was M6. The other relatively abundant metabolites in the brain followed the order M5, M13, M11, and M12. M6, M13, M11, and M12 were relatively more abundant in plasma (Figure 2B).

### 2.3. Pegasus Profoundly Alleviates Anxiety and Improves Motor Performance in TgA53T Mice

**Figure 3:**
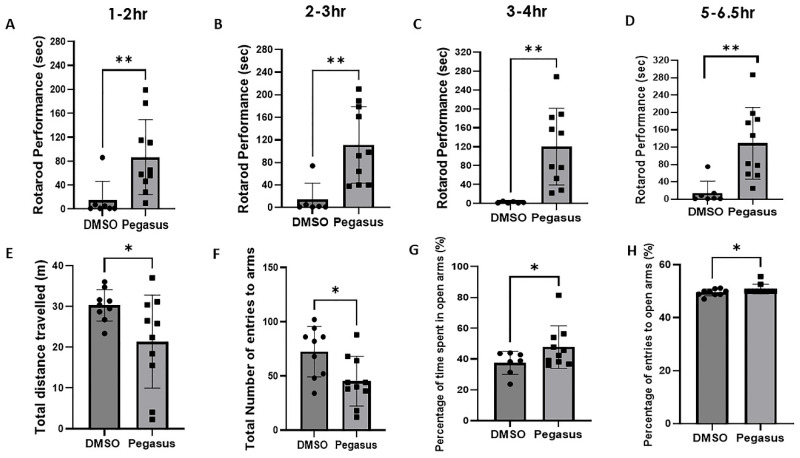
Pegasus profoundly improves anxiety and motor performance up to 6.5 h. Male and female 12–13-month-old TgA53T mice were treated with DMSO or Pegasus at a dosage of 50 mg/kg for 3 weeks. Behavioral assessment started from the 15th day of treatment. (**A**–**D**) The Rotarod test shows that Pegasus treatment improves animal motor performance up to 6.5 h. (**E**) The elevated plus maze test reveals that Pegasus treatment significantly decreases the total distance traveled and the total number of entries to arms (**F**), and significantly increases the percentage of time spent in open arms (**G**) and the percentage of entries to open arms (**H**). Actual *p*-value of the statistical analysis by a two-tailed t test. * *p* < 0.05, ** *p* < 0.01. N = 7–10. All values are presented as mean ± SD.

TgA53T mice (12–13 months old) were treated daily with Pegasus 50 mg/kg i.p. or DMSO for 3 weeks before performing behavioral analysis. A single day assessment (following pre-test training) showed that Pegasus significantly improves motor performance at 1–2 h post-injection (Figure 3A) and continues to improve motor behavior at 2–3 h (Figure 3B), 3–4 h (Figure 3C), and up to 5–6.5 h (Figure 3D) compared to DMSO, suggesting that Pegasus is a potential drug that provides long-lasting effects on motor symptoms. An elevated plus maze test was utilized to assess the anxiety-like behavior of TgA53T mice (12–13 months old). In Pegasus-treated mice, the total distance traveled (Figure 3E) and the total number of entries to arms (Figure 3F) were significantly decreased compared to DMSO. The percentage of time spent in open arms (Figure 3G) and the percentage of entries to open arms (Figure 3H) were also increased in Pegasus-treated mice, suggesting reduced anxiety.

### 2.4. Pegasus Improves Behavioral Deficits of MPTP-Treated Mice

**Figure 4:**
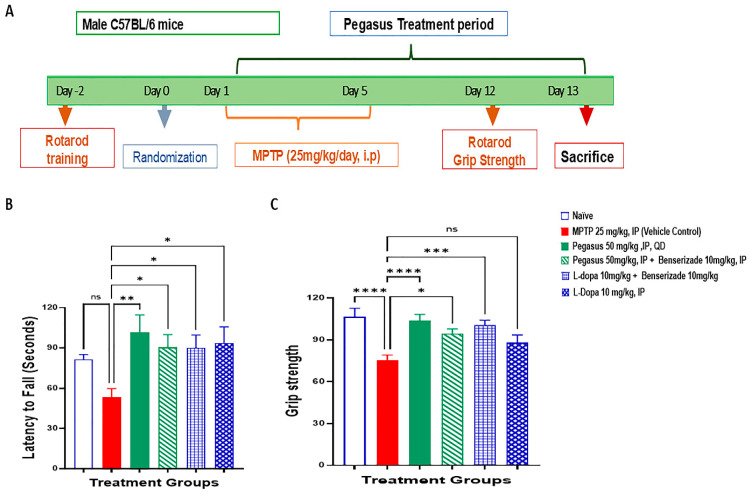
Pegasus improves behavioral deficits of MPTP-induced neuromuscular impairment. The study design (**A**) where C57BL/6 mice are treated with Pegasus and L-dopa alone or in combination with Benserazide significantly increases the latency to fall in the Rotarod test (**B**) and increases the grip strength in the grip strength test (**C**) compared with MPTP vehicle control. The asterisk indicates static significant difference. ns: not significant, * *p* < 0.05, ** *p* < 0.01, *** *p* < 0.001, **** *p* < 0.0001 compared to vehicle. One-way ANOVA; Dunnett’s multiple comparisons test; N = 11–15. All values are presented as mean ± SD.

MPTP was injected to male C57BL/6 mice for 5 consecutive days (Figure 4A) to destroy DA neurons, and then the mice were treated with Pegasus alone or Pegasus + Benserazide compared to L-dopa alone or L-Dopa + Benserazide. Pegasus- and L-Dopa-treated mice had a significantly increased latency to fall, indicating improved motor deficiency induced by MPTP (Figure 4B). Forelimb muscular strength was significantly reversed back to normal levels in Pegasus- and L-Dopa-treated mice (Figure 4C), indicating that Pegasus is sufficient to alleviate the neuromuscular dysfunction induced by MPTP.

### 2.5. Pegasus Reduces Alpha-Synuclein Levels and Increases DA and Its Metabolite HVA Levels in the Brain of TgA53T Mice

**Figure 5:**
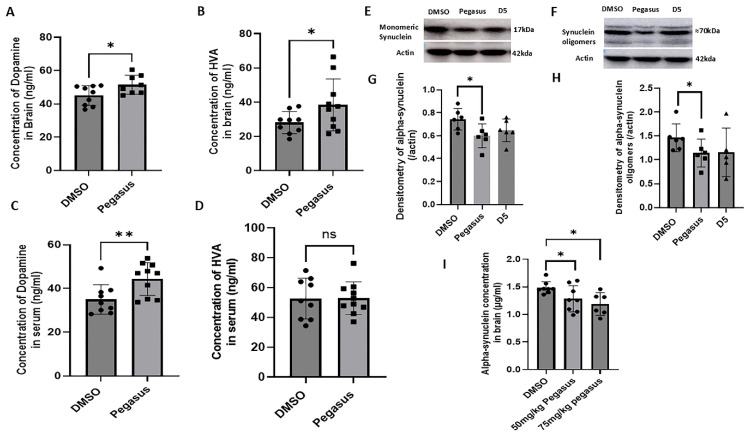
Pegasus reduces alpha-synuclein and increases DA/HVA levels in the brain of TgA53T mice. Old 12–13-month-old TgA53T mice were injected with Pegasus at the once daily dosage of 50 mg/kg or 75 mg/kg for 3 weeks. Pegasus 50 mg/kg significantly increased DA and its metabolite HVA levels in the brain (**A**,**B**) and DA levels in the serum (**C**) but not HVA levels in the serum (**D**). Younger 6–7-month-old TgA53T mice were injected with Pegasus or D5 once daily for 2 weeks. The WB of soluble brain extracts showed (**E**,**F**) monomeric alpha-synuclein and (**G**,**H**) oligomeric alpha-synuclein levels relative to actin in 4–12% Bis-tris Criterion XT precast gel. (**I**). The ELISA analysis of soluble brain extracts reveals alpha-synuclein levels. Actual *p*-value of the statistical analysis by a two-tailed t test. ns: not significant, * *p* < 0.05, ** *p* < 0.01. N = 5–10. All values are presented as mean ± SD.

Pegasus significantly increases the concentrations of DA in the brain (Figure 5A; *p* = 0.0177) and HVA (Figure 5B; *p* = 0.0374), and significantly increases the concentration of DA (Figure 5C; *p* = 0.0057) though not HVA in the serum (Figure 5D; *p* = 0.4896), compared to DMSO in 12–13-month-old mice, indicating that Pegasus may act as a dopamine carrier. The WB of brain samples harvested from young TgA53T mice (6–7 months old) revealed significant decreases in the levels of both monomeric (Figure 5E,G; *p* = 0.0149) and oligomeric alpha-synuclein (Figure 5F,H; *p* = 0.0437) after treatment with Pegasus compared with D5- and DMSO-treated mice. The concentration of CNS alpha-synuclein via ELISA was reduced in the brain lysates of old (11–12-month-old) TgA53T mice treated with Pegasus compared to DMSO-treated controls (Figure 5I).

### 2.6. Pegasus Significantly Increases the Number of DA-Producing Neurons in the SN and Striatum

**Figure 6:**
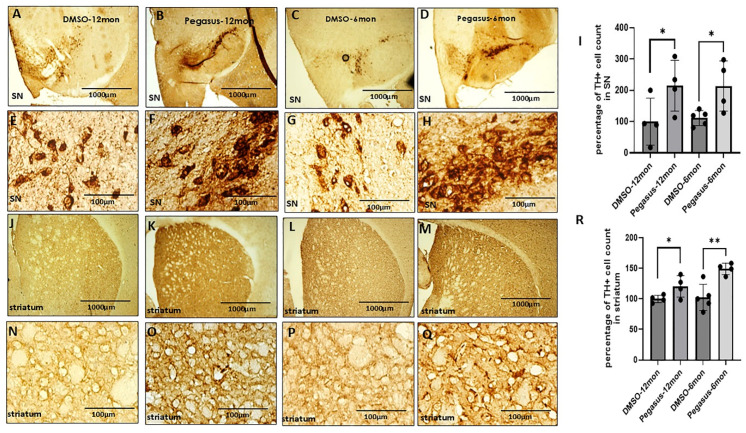
Pegasus significantly increases dopamine neurons in substantia nigra and striatum in TgA53T mice. Here, 12–13-month-old TgA53T mice were injected with Pegasus at the once daily dosage of 50 mg/kg for 3 weeks and 6–7-month-old TgA53T mice were injected with Pegasus at the once daily dosage of 10/25/50 mg/kg i.p. for a total of 2 weeks. The DAB staining of TH shows that Pegasus significantly increases the TH+ cell count in the substantia nigra and striatum in both 12–13-month-old TgA53T mice (**B**,**F**,**K**,**O**) and 6–7-month-old TgA53T mice (**D**,**H**,**M**,**Q**) compared with the corresponding DMSO-treated TgA53T mice ((**A**,**E**,**J**,**N**) and (**C**,**G**,**L**,**P**)). The results were verified by the quantification of the percentage of TH+ cell count in both the SN (**I**) and the striatum (**R**). Actual *p*-value of the statistical analysis by a two-tailed t test. * *p* < 0.05, ** *p* < 0.01. N = 4–5. All values are presented as mean ± SD.

To further examine whether Pegasus has any effects on DA-producing neurons, we performed immunohistochemistry to visualize tyrosine hydroxylase (TH+)-positive neurons in both the SN and striatum in Pegasus-treated versus untreated mice. TH is a rate-limiting enzyme in the biosynthesis of DA in the brain. We used DAB staining to highlight the TH+ neurons. Treatment with Pegasus dramatically increases the cell count of TH+ neurons in the SN (Figure 6A–I) and DA nerve terminals in the striatum (Figure 6J–R) in both young (6–7-month-old) and old (12–13-month-old) TgA53T mice compared to DMSO. This suggests that Pegasus is an effective agent for neuroprotection against DA deficiency.

### 2.7. Pegasus Reduces Astrocytic Staining in TgA53T Mice

**Figure 7:**
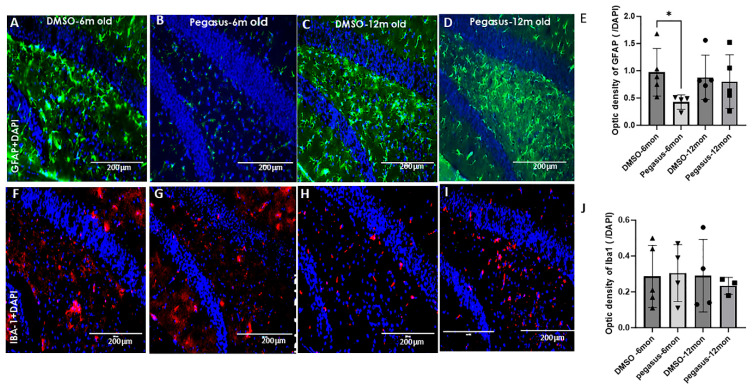
Pegasus reduces astrocytic reactivity in TgA53T mice. GFAP staining in the hippocampus reveals a decrease in the immunoreactivity of astrocytes in young (6–7-month-old) TgA53T mice treated with Pegasus 50 mg/kg i.p. (**B**,**E**) compared to mice treated with DMSO (**A**,**E**) but not in old (12–13-month-old) mice (**C**–**E**). IBA-1 staining in the hippocampus shows no immunoreactivity difference in microglia in young (6–7-month-old) (**F**,**G**,**J**) and old (12–13-month-old) (**H**–**J**) TgA53T mice. Actual *p*-value of the statistical analysis by a two-tailed t test. * *p* < 0.05. N = 4–5. All values are presented as mean ± SD.

Neuroinflammation is one of the most important pathogeneses in PD in addition to alpha-synuclein aggregation [9,10,11,12]. To examine the effects of Pegasus on glial cell activation, we performed immunohistochemistry of astrocyte and microglia from the brains of treated vs. untreated young (6–7-month-old) and old (12–13-month-old) TgA53T mice. The astrocyte staining with glial fibrillary acidic protein (GFAP) in the hippocampus of young TgA53T mice showed a significant reduction in astrocyte reactivity with Pegasus compared to DMSO in young (Figure 7A,B,E), but not old TgA53T mice (Figure 7C–E). GFAP staining was not appreciably different between treatments in other brain regions, including the SN and cortex. No differences in IBA1 staining were observed in the hippocampus of Pegasus-treated mice compared to DMSO control mice (Figure 7F–J).

## 3. Discussion

Pegasus is metabolized into a total of thirteen metabolites, which were observed in rat brain and plasma. Many of the metabolites were formed after the amide cleavage of the parent, Pegasus, into the tetracycline or the DA scaffold and further biotransformation of those scaffolds. In rat brain and plasma samples, the major metabolite was M6, which is a modified tetracycline consisting of C-N bond cleavage + NH_2_ to OH-2H+O. The other relatively abundant metabolites in the brain followed the order M5, M13, M11, and M12. M11 is DA conjugated to cysteine (C N bond cleavage + DA + linker + cysteine) that was abundantly detected in both the plasma and brain, suggesting that the linker ± cysteine serve as stabilizers to protect DA from rapid plasma oxidation and breakdown and delivery into the brain at high relative brain–plasma abundance (130%). Pegasus enters the brain as the mouse studies showed (Figure 1D,E,F), albeit less than 1% is detected in the mouse and rat brain. However, further in-depth studies in rats showed that Pegasus is metabolized into 13 fragments (Figure 2B, table) and 2 of these fragments are DA + linker (M8) and DA + linker + cysteine (M11). Both M8 and M11 are abundantly detected in the brain and the blood in rats and the relative abundance of brain–plasma is 318 and 131%, respectively. These data suggest that Pegasus may either act as a “pro-drug” that is metabolized in the periphery (perhaps the liver) and yields DA that is stabilized via linker ± cysteine and these metabolites are in turn delivered to the brain, or intact Pegasus may directly enter the CNS where it is finally metabolized. Indeed, more data indicate that, under physiological conditions, 2–6% of all small molecules can cross the BBB intact [8,13]. Collectively, these findings suggest that the ability of Pegasus to yield neuropathological effects in PD models may be either due to its ability as an intact molecule to enter the brain where it can be metabolized, or the possibility of peripheral multiple liver passages and phases of metabolism that can increase the level of its metabolites. Furthermore, Pegasus is moderately bound to mouse, rat, dog, and human plasma (Avg. 91–98%) using the equilibrium dialysis method.

The abundance of the DA–cysteine metabolite in plasma and its detection in the brain is in stark contrast with the highly challenging delivery of peripheral levodopa. When levodopa is administered alone, only 1% of each oral dose can reach the brain with a short half-life (T_1/2_ 0.77–1.08 h) [13]. However, various formulations of levodopa in combination with dopa-carboxylase enzyme inhibitor (DDCI) can enhance levodopa brain entry up to 10% with extended T_1/2_ 1–1.5 h [14]. When levodopa is administered with DDCI and selective catechol-O-methyl transferase (COMT) inhibitors, it may reach up to 40% in the brain [15]. Our PK data indicate that the half-life of Pegasus is extended longer than 4 h (T_1/2_ 4.11) in plasma, and despite its low brain/serum ratio (0.7%), the T_1/2_ in the brain is also increased to 3.77 h. We observe a highly desirable total drug exposure over time as indicated by the area under the curve (AUC 17,164 ng × h/mL), suggesting that the metabolism of Pegasus via phase I and phase II provides extended-release time (or slower clearance) of the M11 metabolite, resulting in prolonged and improved performance on Rotarod in TgA53T mice. Importantly, the short plasma T_1/2_ of existing levodopa formulations is manifest in the short duration of the antiparkinsonian response that parallels the plasma levodopa levels and their numerous side effects. PD patients may need multiple dosing (up to 10 a day) of levodopa and its various formulations [16] due to the shrinkage of the therapeutic window as the disease progresses [17,18]. Long-lasting drug effects or slower clearance may help reduce dosing frequency and improve patient adherence [6,19]. Pegasus may prolong the antiparkinsonian response due to the abundance of brain penetration of M11 and its plasma levels, which may result in the prolongation of the duration of its action, as seen in TgA53T mice that continued to perform well on Rotarod up to 6.5 h. Additionally, the prolongation of t_1/2_ of dopaminergic therapy due to reduced dopamine breakdown (e.g., with monoamine oxidase inhibitors) may attenuate motor fluctuations and overcome the complications of long-term therapy with levodopa. Motor fluctuations could predictably improve with DA bioavailability. Long-term complications are attenuated by an early combination of levodopa with DA agonists [20], and the metabolism of Pegasus into DA, that may act as a DA receptor agonist [7], can potentiate the action of Pegasus and perhaps offer a wider therapeutic window.

Previous studies in vitro and in vivo demonstrated that a tetracycline derivative that crosses the BBB inhibited α-synuclein aggregation via the potential dismantling of α-synuclein fibrils into smaller fragments to prevent the seeding of alpha-synuclein and reduce inflammation [21,22,23,24]. These findings agree with our data that Pegasus reduces monomeric and oligomeric alpha-synuclein in TgA53T mice and suppresses astrocytic activity, suggesting an anti-inflammatory response, leading to the improvement of motor and behavioral outcomes in these mice. It is unlikely that Pegasus’ effect on DA numbers is via neurogenic mechanisms; however, the increase in DA levels may be due to a neurotrophic effect due to the reduction in alpha-synuclein oligomerization and the possible scavenging of reactive oxygen species [21,22,23,24]. The relative high abundance of tetracycline metabolites of Pegasus (M6, M10, and M12) in the brain indicates that these molecules may be responsible for the reduction in brain alpha-synuclein levels [25]. It has been suggested that the aggregation of alpha-synuclein can induce glutamate release that can lead to excitotoxic effects, but DA may dampen glutamate excitotoxicity [24,26]. Our data indicate a simultaneous decrease in alpha-synuclein aggregation and DA increase in TgA53T mice in association with the attenuation of astrocytic activity that is responsible for the detoxification of glutamate. Tetracyclines are generally regarded as anti-oxidants and they represent a class of antibiotics in animal models and human disease [27,28]. Minocycline prevents striatal cell loss in MPTP models of PD [29], and our data show a reduction in cell death in TgA53T mice and the recovery of DA and its metabolite, HVA [30], both in plasma and the brain. Pegasus also counters the effects of the destruction of DA neurons with the chronic administration of MPTP and leads to behavioral improvement. Taken together, these data suggest that Pegasus exerts a multifunctional role due to its ability to deliver DA into the brain, where it may act as a DA agonist [7] and reduce the seeding and oligomerization of alpha-synuclein and inflammation.

In conclusion, Pegasus provides a mechanistic strategy that concurrently lowers alpha-synuclein and neuroinflammation, replenishes DA loss, and acts as a DA receptor agonist. Pegasus may effectively target multiple phenomena associated with PD pathology. Although the capsase-3 studies showed no apparent toxicity in organs and 50 mg/kg was a tolerated dose in mice with no visible side effects, the fragmentation and metabolism of Pegasus into 13 metabolites, albeit beneficial for the release and delivery of stable DA to the brain, must be further investigated through in vivo toxicity, pharmacology, and toxicokinetic studies to determine the safety of these molecules and their bioavailability via different routes of administration. Furthermore, levodopa absorption is quite variable from individual to individual in PD, and the administration and optimization of peripheral levodopa to PD patients to reduce side effects and improve antiparkinsonian effects are very challenging. The enhanced bioavailability and exposure of Pegasus in plasma and its ability to deliver DA into the brain may help overcome the challenges of levodopa dosing, including the frequency of administration. Future human studies may help delineate the potential pro-drug effect of Pegasus on DA levels.

## 4. Materials and Methods

### 4.1. Pharmacokinetics

The formulation vehicle of Pegasus was 5% DMSO + 35% PEG400 + 60% normal saline. Pharmacokinetics (PK) was investigated after a single i.p. injection of 50 mg/kg or 100 mg/kg Pegasus to male and female 5–6-month-old wild-type C57BL/6 mice. The animals were euthanized with CO2 (3.7 L/min) in a mouse chamber (6.5″ × 12.5″ × 5.5″), equivalent to approximately 50% flow rate per minute, and death was verified by cervical dislocation. The brains were washed with normal saline for 1 min and then perfused with 4% paraformaldehyde (PFA) for 15 min. The blood was collected via cardiac puncture. The separation of the benzoyl derivatives was achieved on a column Luna Omega C18 PS, 150 × 2.1 mm, 1.6 μm (Phenomenex, Torrance, CA, USA). Pegasus concentrations in serum and brain were measured by UHPLC-MS/MS. Mobile phase A was water, 0.1% (*v*/*v*) formic acid (FA), and 6.7 mM ammonium formate. Mobile phase B was acetonitrile and 0.1% formic acid. Gradient elution (min—%A/%B): 0—98/2; 0.2—98/2; 6—30/70; 7.5—30/70; 7.6—98/2; 8.2—98/2; the flow rate was 400 μL/min.

### 4.2. TgA53T Mouse Experiment

Male and female (12–13 months) transgenic mice that harbor the arginine to threonine (A53T) mutation of human alpha-synuclein under the control of prion promoter [31] were treated with an i.p. injection of Pegasus 50 mg/Kg or 75 mg/Kg or DMSO daily for 3 weeks. The animals were sacrificed with CO_2_. All experiments were conducted in full compliance with the recommendations of Georgetown University Animal Care and Use Committee.

### 4.3. Dose Escalation Study and Tissue Toxicity via Caspase-3 Activity

Tissue toxicity was assessed in young (6–7-month-old) and old (12–13-month-old) A53T mice. The mice received daily i.p. escalating doses of Pegasus or control D5 (tetracycline derivative with no DA) vs. DMSO of 10, 25, 50, and 100 mg/kg for 4 days with each dose up to 16 days, and tissues were analyzed via caspase-3 activity. The caspase-3 activity assay (EnzChek^®^ Invitrogen, Waltham, MA, USA) was conducted according to manufacturer’s protocols as we previously described [32,33].

### 4.4. In Vivo Metabolism

Metabolite profiling was performed in rat brain and plasma samples to detect phase-I and phase-II metabolites in male Sprague Dawley rats (6–8 weeks old, n = 6) injected (i.p.) with 50 mg/kg Pegasus. The formulation vehicle of Pegasus was 5% DMSO + 35% polyethylene glycol (PEG) 400 + 60% normal saline, and this formulation was used to measure the plasma and brain concentrations of Pegasus and all other metabolites. All samples obtained after Pegasus dosing in rats by the i.p. route of administration were used for metabolite profiling and compared to normal saline. Blood (n = 16 samples) was collected via retro-orbital plexus puncture 0.25, 1, 4, 6, 8, and 24 h pre-dose, and brain sampling was 1, 4, and 24 h post-dose (n = 6 per group). The brain samples were cleaned and perfused, then washed with PBS. The brains (approximately 1.5 g) were transferred to a 50 mL centrifuge tube and 36 mL of ice-cold PBS was added. The mixture was homogenized for about 1–2 min to obtain a uniform suspension. The precipitate was allowed to settle for 30 min at 4 °C, and 15 mL of the middle layer was collected. The plasma and brain samples were processed by the protein precipitation method. In total, 175 μL of acetonitrile was added to 25 μL of plasma samples. The samples were mixed, followed by centrifugation at 4000 rpm for 20 min. The supernatant from the samples was subjected to LC-MS/MS analysis.

The samples were analyzed by liquid chromatography tandem mass spectrometry (HPLC: LC series, Shimadzu, Chiyoda-ku, Tokyo, Japan; mass spectrometer: API4000 Triple Quad mass spectrometer, Applied Biosystems, Carlsbad, CA, USA). The samples (n = 6) were processed using protein precipitation and 1 mM stock solution, prepared in DMSO, spiked into acetonitrile to a final concentration of 4 µM, and processed using liquid chromatography tandem mass spectrometry, employing a linear gradient method using MH+/MH−(Q1) and product ion (MS/MS) scans. The evaluation of the Pegasus metabolites included demethylation, de-hydroxylation, amide cleavage, oxidation, de-hydrogenation, glucuronidation, sulfation, glutathione conjugation, and several other possibilities. The relative abundance of Pegasus and its metabolites in the brain and plasma was determined at the ionization parameters of the parent.

### 4.5. MPTP Experiment

During the experimental period, animals were housed in a single experimental room (5 per cage in standard cages) and weighed. On randomization day, the corresponding body weights were recorded, and intra-group variations did not exceed 15% of the mean body weight. The study was performed at an in-house facility registered by the Committee for the Control and Supervision of Experiments on Animals (CCSEA). The registration was in accordance with the protocol approved by the Institutional Animal Ethics Committee (IAEC) of Eurofins, India. All the ethical practices laid down in the CCSEA guidelines for animal care were followed during the conduct of the study. Furthermore, the procedures used in this study plan were designed to conform to the accepted practices and to minimize or avoid the risk of causing pain, distress, or discomfort to the animals. The number of animals that were selected for use in this study was considered the minimum requirement to meet rational scientific endpoints for this type of study.

### 4.6. Administration of MPTP

From day 1 to day 5, MPTP was administered to all the groups except Naive at a dose of 25 mg/kg, i.p., QD. Treatment with Pegasus alone (50 mg/kg, i.p., BID), a combination of Pegasus 50 mg/kg, BID + Benserazide 10 mg/kg, QD, or a combination of L-Dopa 10 mg/kg, QD + Benserazide 10 mg/kg, QD or standalone L-Dopa 10 mg/kg, QD, started on day 1 and continued until day 13. On day 13, after the first dose of Pegasus, 4 animals/time point from each group were sacrificed at 0.5, 1, 3, 6, and 12 h, and the blood and brains were collected.

### 4.7. Rotarod Test

The mice were placed on an accelerating Rotarod (Columbus Instruments, Columbus, OH, USA) equipped with individual timers for each mouse. The mice were trained to stay on the rod at a constant 5 rpm rotation for at least 5 min, and then the speed gradually increased by 0.2 rpm/min and the latency to fall was measured. The mice were trained for 3 consecutive days before the final measurement.

### 4.8. Elevated Plus Maze Test

The mice were placed individually in the center of an elevated, plus-shaped (+) apparatus with two open and two closed arms. The number of entries to open arms, the number of entries to closed arms, the time spent in open arms, the time spent in closed arms, the total distance traveled, and the total number of entries to both open and closed arms during a 10 min period were recorded by trained and blinded observers.

### 4.9. Grip Strength Test

The maximal muscle/grip strength of forelimbs was measured using a strength meter. Each mouse was made to hold the grid only by its forepaws, while gently being pulled back by its tail, and the maximal grip strength value displayed was recorded.

### 4.10. Immunohistochemistry

Animals were deeply anesthetized with a mixture of Xylazine and Ketamine (8:80 mg/kg), washed with normal saline for 1 min, and then perfused with 4% paraformaldehyde (PFA) for 15 min. The brains were dissected out and immediately stored in 4% PFA for 24 h at 4 °C, and then transferred to 30% sucrose at 4 °C for 48 h. The brains were cut using a cryostat microtome into 20 µm thick coronal sections and stored at −20 °C. Staining for tyrosine hydroxylase (TH) was probed with anti-tyrosine hydroxylase antibody (Cat No: AB152, Sigma-Aldrich, St. Louis, MO, USA). GFAP (glial fibrillary acidic protein) was stained with anti-GFAP monoclonal antibody (Cat No:MS1376P1, Fisher Scientific, Newington, NH, USA). Nuclear staining with 4′,6-diamidino-2-phenylindole (DAPI) was performed according to the manufacturer’s protocol (Life Technologies, Miami, FL, USA). TH^+^ cells were counted in a stereology-like manner across multiple visual fields (typically 20 fields) in a single animal, and the same procedure was repeated on several animals to obtain the best approximation of TH staining.

### 4.11. Western Blot

To extract the soluble proteins, whole brain tissues, which excluded the olfactory bulb, cerebellum, and spinal cord, were isolated and homogenized in 1 × STEN buffer (50 mM Tris (pH 7.6), 150 mM NaCl, 2 mM EDTA, 0.2% NP-40, 0.2% BSA, 20 mM PMSF, and protease cocktail inhibitor). The proteins were quantified and normalized for WB (and ELISA) on 4–12% Criterion™ XT Bis-Tris Protein Gel (Cat No: 3450125, Bio-rad, Hercules, CA, USA). Beta-actin (β-actin) was probed (1:3000) with monoclonal antibody (Cat No: MAB1501R, Emdmillipore, Danvers, MA, USA). Human alpha-synuclein was probed (1:4000) with monoclonal antibody (Cat No: AHB0261, Thermo Fisher, Waltham, MA, USA). Human oligomeric alpha-synuclein was probed (1:500) with anti-alpha-synuclein oligomer-specific syn33 antibody (Cat No: ABN2265, Sigma-Aldrich, St. Louis, MO, USA). WBs were quantified by densitometry using Quantity One 4.6.3 software (Bio Rad, Hercules, CA, USA) and Image J version 1.54m.

### 4.12. ELISA

The levels of DA and its metabolite HVA were measured using a commercially available ELISA (Cat No. MBS269234 and Cat# MBS1601074, MyBiosource, San Diego, CA, USA) on serum and brain tissue soluble extracts. Human alpha-synuclein was measured via a human-specific ELISA (Cat# 448607, BioLegend, San Diego, CA, USA) according to the manufacturer’s protocol.

### 4.13. Statistical Analysis

All statistical analyses were performed using GraphPad Prism version 10 (GraphPad software, Inc, San Diego, CA, USA). One-way analysis of variance (ANOVA) (and nonparametric or mixed) tests were used in the comparison of means of multiple groups. A two-tailed Student’s t test (and nonparametric tests) was used in the comparison of means of two groups. The Shapiro–Wilk test was used for checking normality before performing statistic tests. Asterisks denote actual *p*-value significances (* *p* < 0.05, ** *p* < 0.01, *** *p* < 0.001 and **** *p* < 0.0001), and N is the number of animals or the number of independent experiments (cell cultures) per group. Unless otherwise indicated, data are expressed as mean ± SD.

## Data Availability

The final data generated and analyzed, materials, and all interpretations are available to the scientific and non-scientific community upon request (to C.M.). No datasets are deposited in a repository and there is no web link to any datasets.

## Supplementary Materials

The following supporting information can be downloaded at: https://www.mdpi.com/article/10.3390/ijms26094251/s1.

## Abbreviations

The following abbreviations are used in this manuscript:

PD	Parkinson’s disease
ELISA	Enzyme-linked immunosorbent assay
i.p.	Intraperitoneal
CNS	Central nervous system
PK	Pharmacokinetics
DA	Dopamine
MPTP	1-methyl-4-phenyl-1,2,3,6-tetrahydropyridine
SN	Substantia nigra
BBB	Blood–brain barrier
A53T	Alanine to threonine mutations
Tg	Transgenic
TH	Tyrosine hydroxylase
DMSO	Dimethylsulfide
HVA	Homovanillic acid
