# Supplementary figures and images for: A Novel Small Molecule Enhances Stable Dopamine Delivery to the Brain in Models of Parkinson’s Disease
Source: Int J Mol Sci. 2025 Apr 30;26(9):4251. doi: 10.3390/ijms26094251 (PMC12072186; doi:10.3390/ijms26094251)

## Slide 1
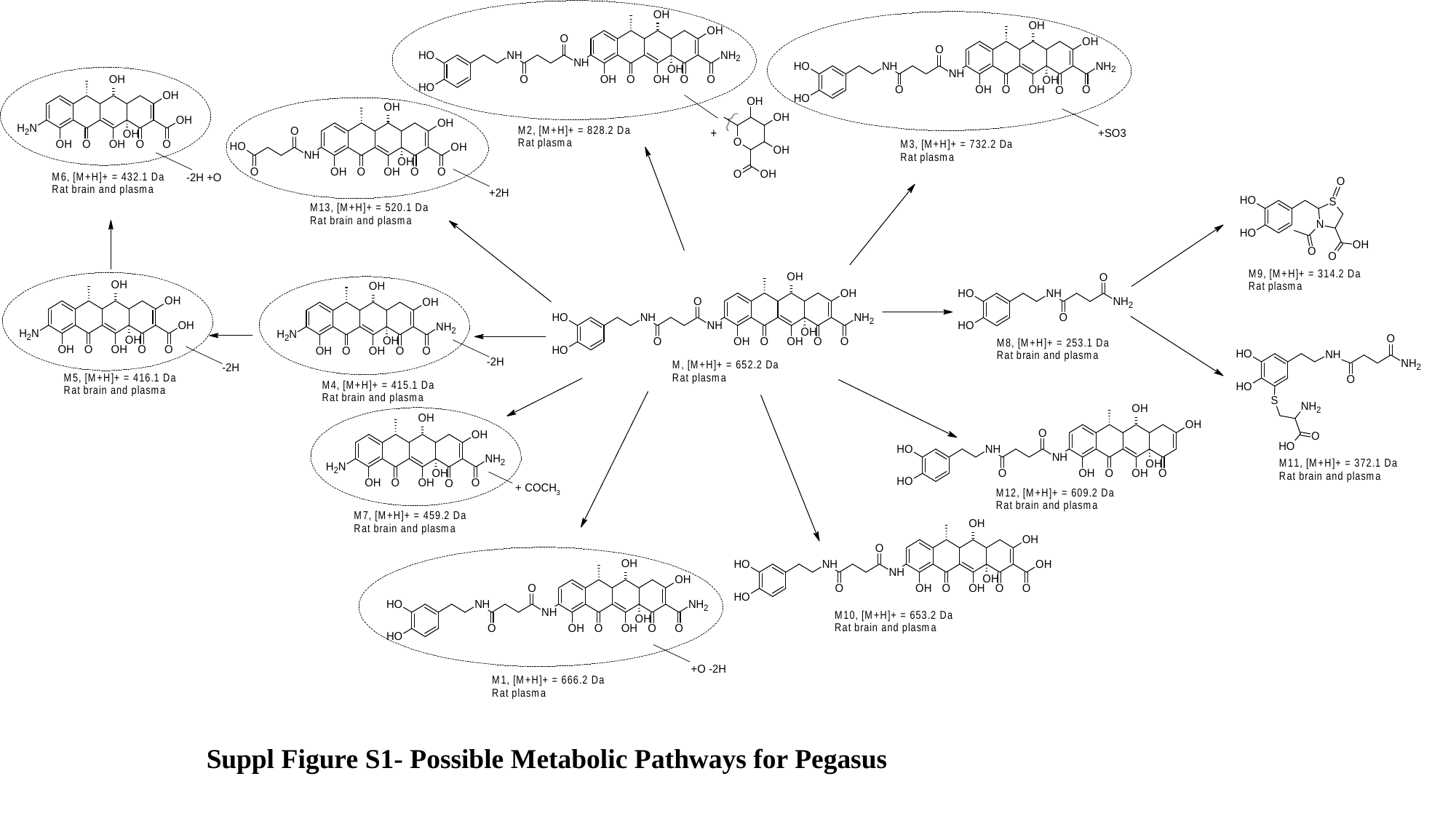

Suppl Figure S1- Possible Metabolic Pathways for Pegasus

Supplement: Supplementary file 1 [file ijms-26-04251-s001.zip › ijms-3551837-supplementary.pptx]
